# Development and Evaluation of *Ginkgo biloba*/Sodium Alginate Nanocomplex Gel as a Long-Acting Formulation for Wound Healing

**DOI:** 10.3390/gels8030189

**Published:** 2022-03-19

**Authors:** Shadab Md, Samaa Abdullah, Nabil A. Alhakamy, Rasheed A. Shaik, Basmah Medhat Eldakhakhny, Ulfat Mohammad Omar, Basma G. Eid, Akhalakur Rahman Ansari, Abdulmohsin J. Alamoudi, Waleed Y. Rizg, Yassine Riadi, Sunil Pazhayanur Venkateswaran, Md Abdur Rashid

**Affiliations:** 1 Department of Pharmaceutics, Faculty of Pharmacy, King Abdulaziz University, Jeddah 21589, Saudi Arabia; nalhakamy@kau.edu.sa (N.A.A.); wrizq@kau.edu.sa (W.Y.R.); 2 Center of Excellence for Drug Research & Pharmaceutical Industries, King Abdulaziz University, Jeddah 21589, Saudi Arabia; ajmalamoudi@kau.edu.sa; 3 Mohamed Saeed Tamer Chair for Pharmaceutical Industries, King Abdulaziz University, Jeddah 21589, Saudi Arabia; 4 Department of Biological Sciences, Faculty of Science, King Abdulaziz University, Jeddah 21589, Saudi Arabia; 5 Department of Pharmacology and Toxicology, Faculty of Pharmacy, King Abdulaziz University, Jeddah 21589, Saudi Arabia; rashaikh1@kau.edu.sa (R.A.S.); beid@kau.edu.sa (B.G.E.); 6 Department of Clinical Biochemistry, Faculty of Medicine, King Abdulaziz University, Jeddah 21589, Saudi Arabia; beldakhakhny@kau.edu.sa; 7 Department of Biochemistry, Faculty of Sciences, King Abdulaziz University, Jeddah 21589, Saudi Arabia; uomer@kau.edu.sa; 8 Princess Dr. Najla Bint Saud Al- Saud Center for Excellence Research in Biotechnology, King Abdulaziz University, Jeddah 21589, Saudi Arabia; 9 Center of Nanotechnology, King Abdulaziz University, Jeddah 21589, Saudi Arabia; akhalakurkau@gmail.com; 10 Department of Pharmaceutical Chemistry, College of Pharmacy, Prince Sattam Bin Abdulaziz University, Al-Kharj 11942, Saudi Arabia; y.riadi@psau.edu.sa; 11 Department of Pathology, School of Medicine, International Medical University, Bukit Jalil, Kuala Lumpur 57000, Malaysia; sunil.pazhayanur@gmail.com; 12 Department of Pharmaceutics, College of Pharmacy, King Khalid University, Abha 62529, Saudi Arabia; mdrashid@kku.edu.sa

**Keywords:** sodium alginate, *Ginkgo biloba* extract, gels, nanocomplex, alginate topical delivery

## Abstract

The aim of the study was to develop and evaluate the *Ginkgo biloba* nanocomplex gel (GKNG) as a long-acting formulation for the wound healing potential. Pharmaceutical analysis showed an average particle size of 450.14 ± 36.06 nm for GKNG, zeta potential +0.012 ± 0.003 mV, and encapsulation efficiency 91 ± 1.8%. The rheological analysis also showed the optimum diffusion rate and viscosity needed for topical drug delivery. Fourier transform infrared spectroscopy (FTIR), powder X-ray diffractometry (PXRD), scanning electron microscopy (SEM), and transmission electron microscopy (TEM) analysis further confirmed the success of GKNG. The in vivo study showed increments in the antioxidant enzymes superoxide dismutase (SOD) and glutathione peroxidase (GPx) and a lower level of lipid peroxidation (MDA) after GKNG treatment. The GKNG group showed upregulations in collagen type I, as alpha 1 collagen (COL1A1), and collagen type IV, as alpha 1 collagen (COL4A1). Furthermore, the in vivo study showed increments in hydroxyproline, epidermal growth factor (EGF), vascular endothelial growth factor (VEGF), and transforming growth factor-beta 1 (TGF-β1) after the GKNG. Additionally, GKNG effectively increased the wound contraction compared to GK gel and sodium alginate (SA) gel. Based on the in vitro and in vivo evaluation, GKNG effectively accelerated wound healing by modulation of antioxidant enzymes, collagens, angiogenic factors, and TGF-β1.

## 1. Introduction

Skin is the first line of defense of the body and offers a protective barrier between external pathogens, physical injury, and vital internal organs [1]. Thus, healthy skin maintains the appropriate physiological and neurohormonal balance and normal homeostasis [2]. However, the dermal integrity is compromised when exposed to trauma or internal damage, which is termed a wound [1]. A cutaneous wound refers to the loss of functional and anatomical integrity of the epidermal and endodermal layer of skin, whereas wound healing refers to the healing of the skin [3]. Ideally, wound healing begins immediately after the injury and continues for up to years, depending upon various confounding factors [3]. On the other hand, wounds also create a substantial economic burden on the healthcare system. According to the published evidence, more than 0.3 billion people suffer from cutaneous wounds and dysfunctional healing every year. The estimated cost in managing and treating wounds goes beyond USD 10 billion annually [4].

In fact, wound healing is a complex and dynamic process involving inflammation, proliferation, and remodeling and involves multiple cellular, molecular, and immunological factors [5]. Considering the molecular mechanism of wound healing, the roles of various growth factors, cytokines, angiogenic factors, platelets, monocytes, fibroblasts, and growth hormones are pivotal and critical in wound healing [1]. Additionally, cell-cell and cell–matrix interaction, wound contraction, tissue remodeling, re-epithelialization, granulation tissue formation, and angiogenesis, are crucial for wound healing [6]. Besides these factors, persistent oxidative stress in response to tissue injury is a major confounding factor in wound healing [6]. Clinically, wound healing occurs in an anticipated and timely way. Any alteration in the healing mechanism leads to a “keloid scar”, “venous ulcer”, or chronic wound, which is indeed of serious concern and even a life-threatening condition [6]. Thus, it seems reasonable that wound healing is a multifactorial mechanism, and the available therapeutic regimens, bandages, rubbers, and dressing gauze are insufficient to take care of this situation. Moreover, pharmacological therapy for wound healing is costly, leading to pharmacokinetics limitations and hindering the therapeutic outcome.

In recent times, the focus has been shifted towards natural products as a potent therapeutic option for managing and treating wound healing. Since ancient times, natural products or plant extracts have been extensively used to manage and treat various types of skin disorders and wounds. These natural products offer a natural environment for wound healing via antibacterial, disinfectant, debridement, and antioxidant potential. Moreover, these natural products are economical and easy to obtain [1]. The *Ginkgo biloba* leaf extract (GK) is one of the well-established traditional Chinese medicine and consists of various bioactive constituents such as bioflavonoids, ginkgolides, and bilobalide [7]. These bioactive constituents in GK possess potent antimicrobial, antioxidant, and antibacterial properties and could be potential drug candidates in managing and treating wounds [7,8,9].

Therefore, looking into the pharmacological benefit of GK and previously reported studies, topical delivery of GK extract was planned. However, the conventional topical drug delivery has several limitations: reduced permeation, low bioavailability, reduced spreadability, and minimal therapeutic outcome [1,9]. Hence, herein, sodium alginate nanocomplex gel of GK was developed. Nanocomplex gel is a 3D network that consists of hydrophilic polymers such as sodium alginate or chitosan [10]. When used topically, these hydrogels form a physical barrier between the wound and external environment and remove the exudate and hydrate the skin and wound, supporting and accelerating the healing process [11]. Sodium alginate (SA) is a well-known gelling agent and offers higher drug encapsulating properties and extended-release when applied topically. Additionally, SA possesses a proinflammatory and proangiogenic potential that helps in immune cell recruitment and wound healing [12,13,14].

To the best of our knowledge, this is the first attempt to develop a SA nanocomplex gel of GK extract (GKNG). Therefore, we have developed GKNG in the present study and characterized it by rheometric analysis, swelling studies, and gel diffusion test. Moreover, various key parameters for the evaluation of GKNG were performed. Finally, the mechanism and success of GKNG were confirmed by studying its effect on wound contraction, the level of TGF-β, TNF-α, collagens, MMPs, EGF, VEGF, hydroxyproline, the markers of lipid peroxidation (MDA), SOD, and GPx in surgically induced wounds in Wistar rats.

## 2. Results 

### 2.1. Nanocomplex Gel Formulation and Optimization 

Initially, the studied combinations were formed by mixing 30 mg/mL of alginate with ginkgo extract amounts ranging from 10 to 40 mg/mL and were tested for their particle size distribution and Poly Dispersity Index (PDI) values, as shown in Table 1.

The particle size analysis for all combinations showed that Combination 1 was the optimum nanocomplex gel having the lowest size (450.14 ± 36.06 nm), narrowest size distribution, and a reasonable PDI value of 0.32 ± 0.12. This is due to the highest alginate ratio used and alginate shearing and coating force during ultrasonication for Combination 1 compared to the others. Furthermore, the nanoparticles formed could be due to the interaction and complexation that occurred between the ginkgo extract and the alginate gel, which was investigated in the Fourier transform infrared spectroscopy (FTIR) (Thermo-Scientific, NicoletiS10, Waltham, Massachusetts, USA) characterization of the gel’s nanoparticles [14]. In addition, the zeta potential of the optimum Combination 1 showed a positive value (0.012 ± 0.003 mV), which might add to the skin adherence as a topical gel for wound healing purposes. The alginate and ginkgo extract hydroxyl and carboxylate groups initiated hydrophilic and hydrogen bond interactions in the core of the nanocomplex gel, leaving the outer surfaces of the nanohydrogel neutral. This result was supported in the FTIR characterization of the NG interactions and surface functional groups [14,15]. After all, the optimum nanocomplex gel encapsulation efficiency was 91 ± 1.8%.

### 2.2. FTIR Characterizations of Nanocomplex Gel

**Figure 1:**
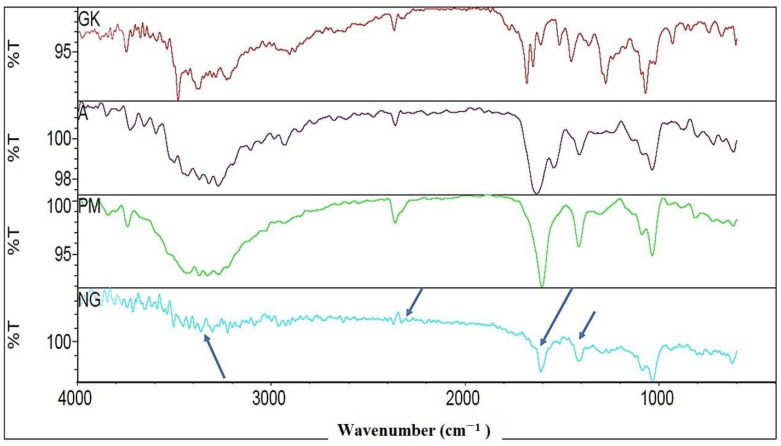
FTIR of *Ginkgo biloba* ginkgo extract (GK), alginate (A), physical mixture of GK and A (PM), and nanocomplex gel (NG).

FTIR spectral analysis of different samples is depicted in Figure 1. GK exhibited characteristic peaks at 1694, 1646, and 1068 cm^−1^. The characteristic peak of 1647 cm^−1^ indicated C=C and C=N stretching, and the peak of 1694 cm^−1^ demonstrated the stretching vibration of C=O. At the same time, the C–OH (alcohols and carboxylic acids) stretching peak appears at 1068 cm^−1^ [9]. The FTIR spectra of alginate, i.e., A (Figure 1), showed characteristics of uronic acid at 987 cm^−1^; a wavenumber of 2965 cm^−1^ demonstrated CH_2_ stretching of alginate, and simultaneously, wavenumbers from 3200 to 3600 cm^−1^ indicated OH stretching [16]. So, FTIR spectra of dry physical mixture of GK and A, i.e., shown as PM in Figure 1, exhibited OH group from 3200 to 3600 cm^−1^, the same as GK and A. It also showed the characteristic peaks at 1694, 1647, and 1068 cm^−1^ for C=N, C=C, and C–OH, respectively, the same as GK and A. On the other hand, the FTIR spectra of nanocomplex gel in Figure 1, i.e., NG, exhibited dissipation of characteristic peaks from 3200 to 3600 cm^−1^, which suggested hydrogen bonding between the OH and CH_2_ group. In addition, the NG spectrum showed lower peaks intensity of 1694, 1646, and 1068 cm^−1^ to support the assumption of the encapsulation of carboxylate and hydroxyl groups in the NG core [15,17].

### 2.3. PXRD Characterizations of Nanocomplex Gel

**Figure 2:**
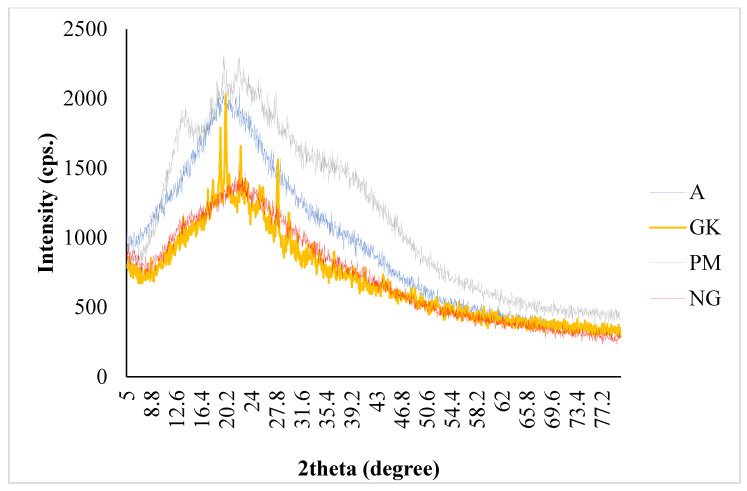
PXRD characterizations of *Ginkgo biloba* ginkgo extract (GK), alginate (A), their dry physical mixture (PM), and nanocomplex gel (NG).

GK extract, alginate (A), their dry physical mixture (PM), and nanocomplex gel (NG) are shown in Figure 2. As per the finding of powder X-ray diffractometry (PXRD) (Maxima XRD-7000X, Shimadzu, Kyoto, Japan), the NG diffractogram appears to be complementary to the GK diffractogram, but the GK diffractogram has some distinctive crystalline peaks compared to the amorphous nature of the NG. The broad peak of the diffractogram indicated the amorphous characteristic of NG, and the crystalline character of GK is confirmed by the presence of a sharp peak in the respective diffractogram. 

### 2.4. SEM Characterizations of Nanocomplex Gel

**Figure 3:**
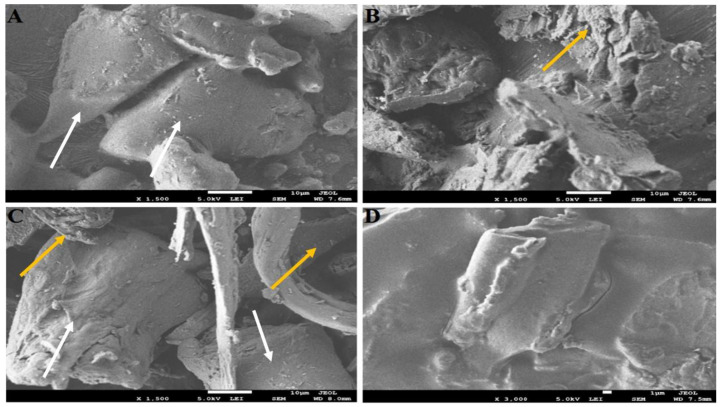
SEM characterization of (**A**) ginkgo extract, (**B**) sodium alginate, (**C**) dry physical mixture, and (**D**) NG. The arrows are indicating the morphology changes between the groups.

The GK extract and alginate (A) morphology were characterized with the irregular powder bed using scanning electron microscopy (SEM) (FEI Inspect F50, FEI, Tokyo, Japan) (Figure 3). Moreover, their dry physical mixture (PM) showed a combination of GK and A morphologies. On the other hand, the optimized nanocomplex gel (NG) confirmed the adsorption and coating that happened between the A and GK during the encapsulation of the NG. Furthermore, the morphologies of the A and GK after the encapsulation in the NG were shown to be more regular (Figure 3A–D).

### 2.5. TEM Characterization of Nanocomplex Gel

**Figure 4:**
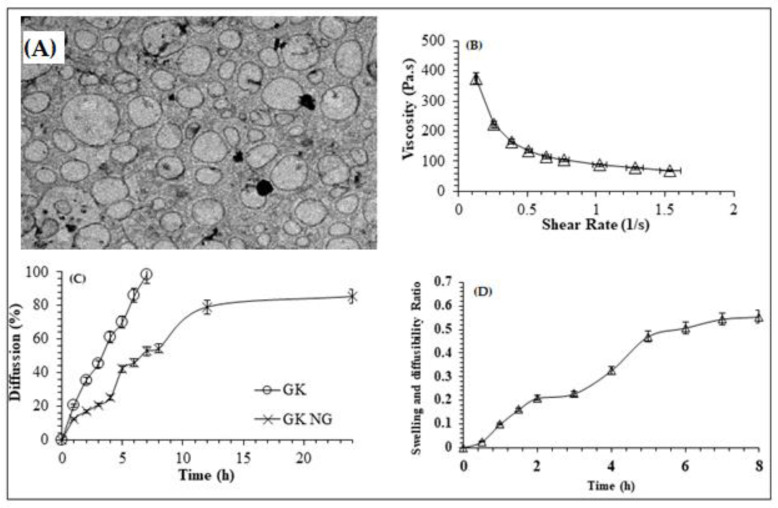
(**A**) TEM and viscosity (**B**) of GKNG. (**C**) Percentage diffusion of free GK and GKNG. (**D**) nanocomplex gel (GKNG) swelling, diffusibility, and resilience test over 8 h.

As shown in Figure 4A, findings of transmission electron microscopy (TEM) (H7500, Hitachi, Tokyo, Japan) imaging showed uniform and spherical-shaped particles with no sign of particle aggregation. The result of the TEM image signifies the homogeneity of formulation and confirms the finding of particle size analysis.

### 2.6. Rheology Study of Nanocomplex Gel

As shown in Figure 4B, the viscosity of the prepared nanocomplex gel changed considerably with respect to the change in shear stress. In other words, increased shear stress reduced the viscosity, and the findings of the study are in accordance with the non-Newtonian or pseudoplastic behavior. As seen in Figure 4B, the highest viscosity was observed at the lowest shear stress. It is further inferred from the findings that the formulated optimized nanocomplex gel follows thixotropic behavior, which means gels can easily be poured after shaking and are viscous upon standing.

### 2.7. Nanocomplex Gel Diffusion Study

For the determination of in vivo drug diffusion behavior, in vitro drug diffusion study is a vital resource. The outcomes of in vitro diffusion study for optimized GKNG are depicted in Figure 4C. After 8 h, the ginkgo diffusion rate for the nanocomplex gel was half that of the raw GK extract. After 24 h, the diffusion rate of the ginkgo raw extract was 0.2-fold more than that of the optimized nanocomplex gel.

Diffusion Rate (mg/h) = Amount diffused (mg)/Time (h)(1)

### 2.8. Swelling, Diffusibility, and Resilience Test

The optimum nanocomplex gel swelling and diffusibility ratio increased by 0.20 ± 0.04 after 2 h of the swelling media exposure compared to the starting/zero-time gel (Figure 4D). Moreover, the nanogel swelling and diffusibility ratio increased by 0.33 ± 0.12 after 4 h of the swelling media exposure compared to the starting/zero-time gel. Interestingly, the optimum nanocomplex gel swelling and diffusibility ratio differences between 5, 6, 7, and 8 h were less than the differences in swelling between the earlier time intervals, indicating a plateau swelling stage [18]. Generally, the swelling profile could be correlated to the water solubilization of the alginate and ginkgo glycosides [15,17]. On the other hand, the plateau stage might indicate that the swelling of the nanogel had a certain extent, and the alginate and ginkgo nanogel preserved a specific resilience degree [15,17].

### 2.9. Effect of Nanocomplex Gel on Antioxidant Parameters

**Figure 5:**
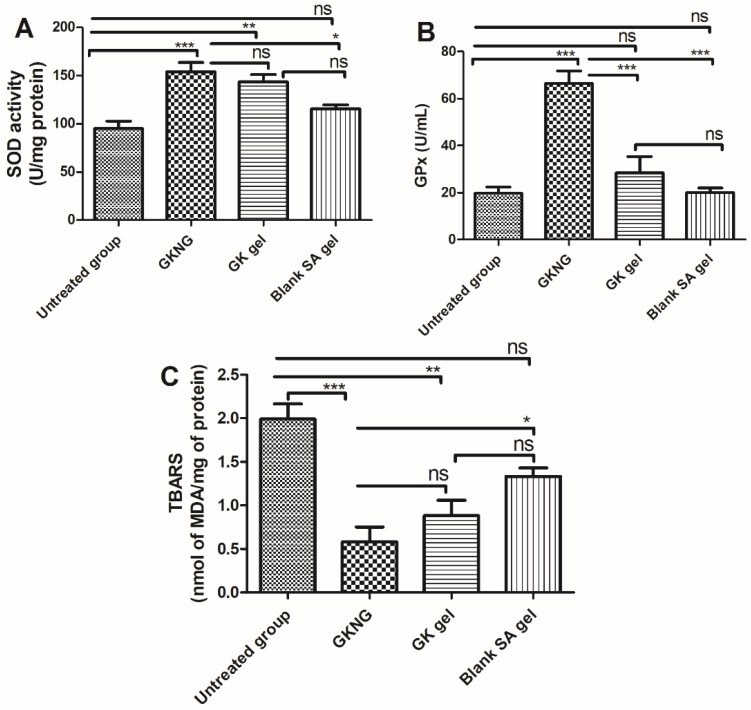
Effects of GKNG, GK gel, and blank SA gel on the markers of antioxidant enzymes (**A**) SOD, (**B**) GPx, and (**C**) MDA. Values are represented as mean ± SD (n = 4). Statistical analysis was performed by one-way ANOVA followed by Tukey’s multiple comparison test. ‘***’, ‘**’, and ‘*’ refer to *p* < 0.001, *p* < 0.01, and *p* < 0.05 and represent significant differences between the treatment and untreated groups. ‘ns’ refers to a nonsignificant difference between the treatment groups and untreated groups.

In the in vivo study, the untreated group showed a significant reduction in SOD and GPx and elevation in the level of MDA (*p* < 0.001), hence signifying the role of increased oxidative stress in the pathogenesis wound. When the animals were treated with GKNG, it significantly increased the enzymatic activity of SOD and GPx and reduced the marker of lipid peroxidation, i.e., MDA (*p* < 0.001 for SOD, GPx, and TBARS). Topical application of ginkgo (GK) gel showed a mild increase in the activity of SOD and GPx (*p* < 0.001), whereas it failed to reduce the level of MDA (a marker of lipid peroxidation). However, topical application of alginate (SA) gel was unable to increase the enzymatic activity of SOD and GPx (*p* > 0.05) and also failed to reduce the level of MDA (*p* > 0.05). Upon comparison of the wound healing potency in lieu of the antioxidant effect of GKNG and GK gel, it was found that both formulations exhibited almost comparable effects, with GKNG being superior. Statistical analysis showed no significant difference between GKNG and GK gel (*p* > 0.05) in increasing the antioxidant activity of SOD and in reducing the level of MDA. When we compared the antioxidant effect of GKNG and GK gel in terms of antioxidant activity of GPx, a superior and significant effect was found for GKNG (*p* < 0.001). Similarly, a significant difference was observed when GKNG was compared with the SA gel for SOD and GPx activity and against the level of MDA (*p* < 0.05, SOD; *p* < 0.001, GPx; *p* < 0.05, MDA) (Figure 5).

### 2.10. Effect of Nanocomplex Gel on Fibrotic Markers

**Figure 6:**
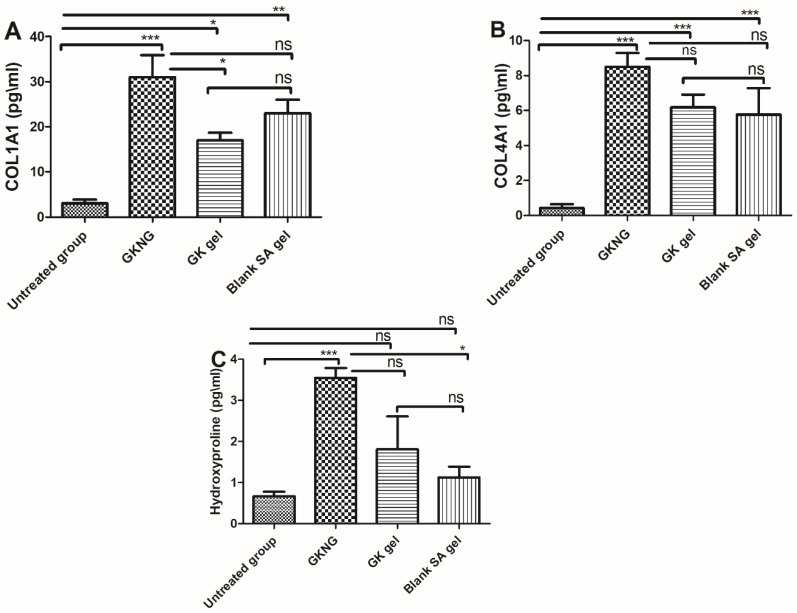
Effects of GKNG, GK gel, and blank SA on the fibrotic markers (**A**) CL1A1, (**B**) CL4A1, and (**C**) hydroxyproline. Values are represented as mean ± SD (n = 4). Statistical analysis was performed by one-way ANOVA followed by Tukey’s multiple comparison test. ‘***’, ‘**’, and ‘*’ refer to *p* < 0.001, *p* < 0.01, and *p* < 0.05 and represent significant differences between the treatment and untreated groups. ‘ns’ refers to a nonsignificant difference between the treatment groups and untreated groups.

In the in vivo study, when the different set of formulations was analyzed for their effect on fibrotic markers, which are crucial for the remodeling phase, the untreated group showed a significant reduction in the levels of COL1A1, COL4A1, and hydroxyproline (*p* < 0.001). When the animals were treated with GKNG, GK gel, and SA gel, the GKNG significantly increased the levels of COL1A1, COL4A1, and hydroxyproline (*p* < 0.001). Topical application of GK gel showed mild elevation in the level of COL1A1 (*p* < 0.05) and significant elevation in the level of COL4A1 (*p* < 0.001) but failed to elevate the level of hydroxyproline (*p* > 0.05). However, topical application of SA gel showed moderate elevation in the level of COL1A1 (*p* < 0.01) and significant elevation in the level of COL4A1 (*p* < 0.001) but failed to elevate the level of hydroxyproline (*p* > 0.05). Upon comparison of the wound healing and fibrotic potency of GKNG and GK gel in terms of increased COL1A1, it was found that GKNG exhibited superior and statistically significant profibrotic potency (*p* < 0.05) compared to the GK gel. However, upon comparing the profibrotic potency of GKNG and GK gel in terms of increased COL4A1 and Hyp, it was found that both formulations exhibited almost comparable effects, with GKNG being superior. Statistical analysis showed no significant difference between GKNG and GK gel (*p* > 0.05) in increasing the levels of COL4A1 and Hyp. Additionally, no difference was observed when GKNG was compared with the SA gel for COL4A1 and COL4A1 (*p* > 0.05), while a significant difference was observed for Hyp (*p* < 0.05) (Figure 6).

### 2.11. Effect of Nanocomplex Gel on Angiogenic Markers and TGF-β1

**Figure 7:**
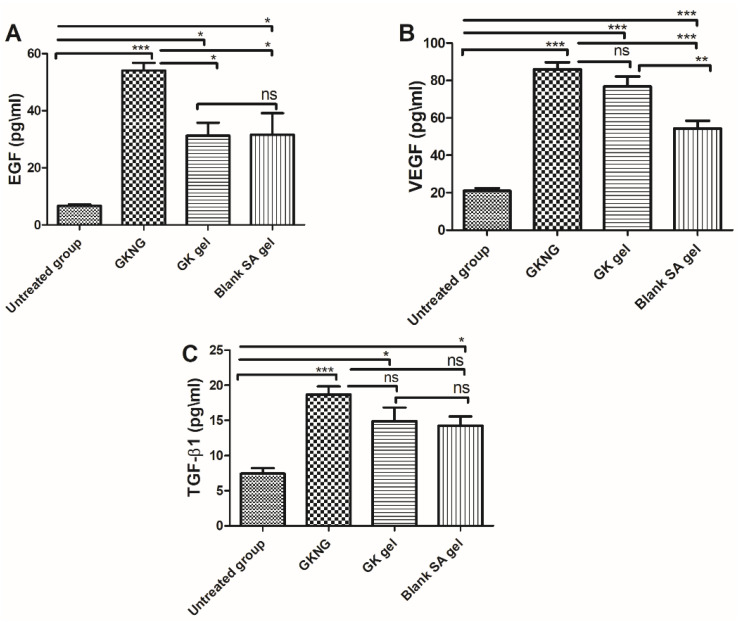
Effects of GKNG, GK gel, and blank SA on the angiogenic markers (**A**) EGF, (**B**) VEGF, and (**C**) TGF-β1. Values are represented as mean ± SD (n = 4). Statistical analysis was performed by one-way ANOVA followed by Tukey’s multiple comparison test. ‘***’, ‘**’, and ‘*’ refer to *p* < 0.001, *p* < 0.01, and *p* < 0.05 and represent significant differences between the treatment and untreated groups. ‘ns’ refers to a nonsignificant difference between the treatment groups and untreated groups.

In the in vivo study, when the different sets of formulations were analyzed for their effects on angiogenic markers and on the level of TGF-β1, which is crucial for the remodeling and contraction phase, the untreated group showed a significant reduction in the level of EGF, VEGF, and TGF-β1 (*p* < 0.001). When the animals were treated with GKNG, GK gel, and SA gel, the GKNG significantly increased the levels of EGF, VEGF, and TGF-β1 (*p* < 0.001). Topical application of GK gel, as well as SA gel, showed mild elevation in the level of EGF (*p* < 0.05), significant elevation in the level of VEGF, and mild elevation in the level of TGF-β1 (*p* < 0.05) Additionally, upon comparing the wound healing and angiogenic activity of GKNG and GK gel in terms of increased EGF level, it was found that GKNG exhibited superior and statistically significant proangiogenic activity (*p* < 0.05) compared to the GK gel. However, upon comparing the angiogenic and profibrotic potency of GKNG and GK gel in terms of increased VEGF and TGF-β1 levels, it was found that both formulations exhibited almost comparable effects, with GKNG being superior. Statistical analysis showed no significant difference between GKNG and GK gel (*p* > 0.05) in increasing the levels of VEGF and TGF-β1. Similarly, when GKNG was compared to SA gel, a significant difference was observed for EGF (*p* < 0.05) and VEGF (*p* < 0.05), whereas no significant difference was observed for TGF-β1 (*p* > 0.05) (Figure 7).

### 2.12. Effect of Nanocomplex Gel on Wound Contraction

**Figure 8:**
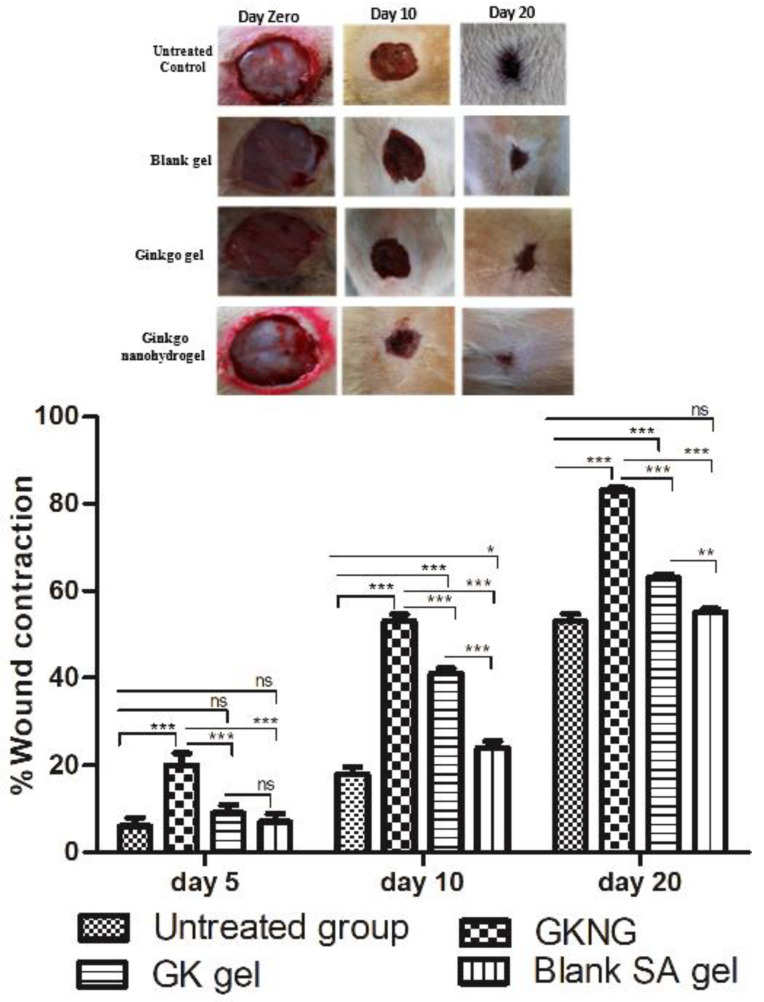
Effects of GKNG, GK gel, and blank SA on the wound contraction. Values are represented as mean ± SD (n = 4). Statistical analysis was performed by one-way ANOVA followed by Tukey’s multiple comparison test. ‘***’, ‘**’, and ‘*’ refer to *p* < 0.001, *p* < 0.01, and *p* < 0.05 and represent significant differences between the treatment and untreated groups. ‘ns’ refers to a nonsignificant difference between the treatment groups and untreated groups.

As depicted in Figure 8, untreated control, GKNG, GK gel, and blank SA gel had an open wound on day zero. However, on days 5, 10, and 20, the topical application of GKNG showed increased wound contraction (closure of wound), followed by the GK gel and blank SA gel when compared with the untreated group. Moreover, the statistical analysis showed a significant difference in the wound closure percentage when compared between untreated and GKNG groups on days 5, 10, and 20 (*p* < 0.001). When the wound closure potential was compared between the GK gel and untreated groups, no significant difference was found on day 5 (*p* > 0.05), whereas a significant difference was observed on days 10 and 20 (*p* < 0.001). However, SA gel showed no significant difference on day 5 (*p* > 0.05), exhibited a significant difference on day 10 (*p* <0.05), and again showed no significant difference on day 20 (*p* > 0.05). Additionally, comparisons between GKNG and GK as well as between GKNG and SA gel on days 5, 10, and 20 showed significant differences in wound closure percentage (*p* < 0.001).

### 2.13. Effect of Nanocomplex Gel on Histopathological Changes

**Figure 9:**
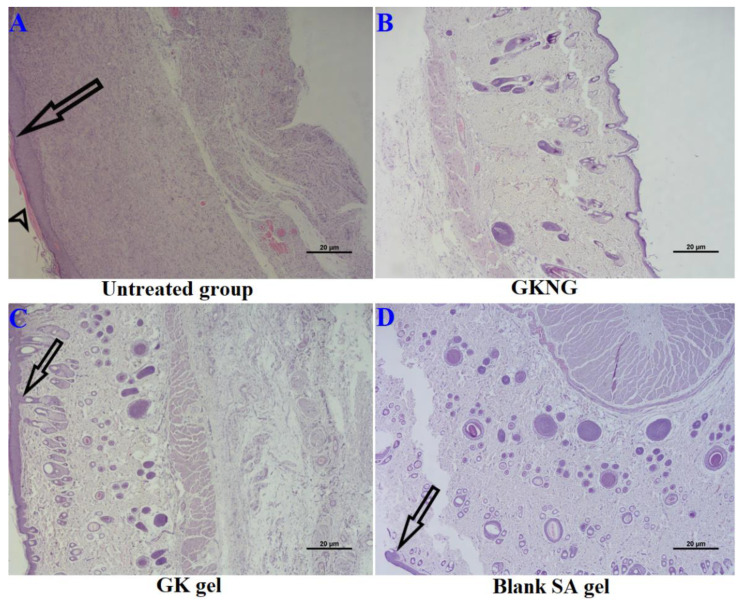
Histopathological analysis of different treatment groups in wound healing on day 20. Subfigure (**A**) is untreated, (**B**–**D**) have been treated with GKNG, GK gel and Blank SA gel. GKNG treatment exhibited the most potent wound healing potential followed by GK gel and Blank SA gel, as evident with a long and short black arrow representing severe acanthosis and focal hyperkeratosis. Scale bar 20 µm at 40×.

As shown in Figure 9, the epidermal layer of the untreated group (Figure 9A) shows severe acanthosis and focal hyperkeratosis. Dermis shows an abundance of collagen fibers with scattered proliferating blood vessels and an extensive chronic inflammatory infiltrate. Features are suggestive of an intact chronic wound. The GKNG-treated group (Figure 9B) shows a much-improved epidermis, dermis, and hypodermis. The epidermis shows no evidence of acanthosis or hyperkeratosis. Dermis shows skin appendages and bundles of collagen fibers. Bundles of muscle fibers are seen. Hypodermis shows subcutaneous tissue and blood vessels. When rats were treated with GK gel and blank SA gel (Figure 9C,D), the epidermis showed moderate acanthosis and no evidence of hyperkeratosis. Dermis shows skin appendages, moderate inflammatory infiltrates, and bundles of collagen and muscle fibers. Hypodermis shows subcutaneous tissue and blood vessels.

## 3. Discussion

A wound is a dermal injury that disrupts the structural and functional integrity of the skin. Whenever there is any injury/wound, the skin heals in an anticipated and predetermined manner [19]. However, any normal physiological healing mechanism alteration results in intractable ulcers or scar formation [3]. The initial phase of wound healing begins with homeostasis and inflammation [20]. Before initiating the healing mechanism, a cascade of blood clotting and vasoconstriction begins, resulting in clot formation that further inhibits blood loss [20].

Additionally, the formed fibrin clot releases chemotactic factors and growth factors and regulates platelet aggregation. Within 24 h of the wound, recruitment of neutrophils occurs and phagocytosis is initiated under the influence of macrophages [21]. Apart from the phagocytic activity, macrophages also produce reactive oxygen species (ROS) that exhibit bactericidal and necrotic activities [22]. In response to neutrophilic recruitment, various growth factors such as TGF-β, EGF, and VEGF; cytokines; extracellular matrix (ECM); and collagens are released, further initiating the proliferation and wound healing [23]. The proliferative phase begins after the inflammatory phase, where angiogenic and growth factors are pivotal. Fibroblast and endothelial cells are important proliferative cells, and during this phase, excessive blood supply (angiogenesis and vasculogenesis) is needed [24]. Therefore, the levels of EGF and VEGF subsequently increase [25]. After proliferation, epithelization begins where migration of keratinocytes occurs, and ultimately, the formation of granulation tissue takes place. In the granulation phase, fibroblast migration to the wound site and its proliferation into the wound occurs. After migration, the synthesis of collagens, such as hydroxyproline, COL1A1, and COL4A1, and fibronectin takes place [26]. The tissue contains granulocytes, macrophages, blood capillaries, collagen, and fibroblasts. The last phase of wound healing is tissue remodeling, where an appropriate balance is established between the formation and degradation of new tissue [19]. At this phase, the process of granulation stops and the maturation of the wound starts. Synthesis of collagen proteins continues in this phase under the influence of COL1A1 and COL4A1, and hydroxyproline and wound contraction begin under the influence of TGF-β1 [19]. TGF-β1 plays a pivotal role in the differentiation of myofibroblasts from fibroblasts; myofibroblasts generate smooth muscle actin and regulate the wound contraction, which is crucial for the closure of wound edge [27]. However, in response to the excessive oxidative stress, reduced level of antioxidant enzymes, and increased MDA level, the normal physiological mechanism of wound healing hampers results in the formation of the chronic wound, which can be fatal [28].

Thus, it is essential to develop a pharmacological strategy to restore normal dermal integrity, support wound healing, and prevent chronic wounds. For this, an optimized long-acting nanocomplex gel of GK extract and sodium alginate (medium molecular weight grade) was prepared based on the particle size analysis. The optimum nanocomplex gel was characterized using the rheometer analysis and the gel diffusion test to assess the ginkgo release at pH 6.8. Furthermore, the formulation nanoparticle arrangements and their interactions were investigated using FTIR, PXRD, SEM, and TEM. These characterizations were conducted to understand the wound healing assay results and analysis of the key for the optimum GKNG, raw GK extract, and alginate groups. Moreover, Bardaa et al. (2021) developed a topical cream of GK extract, and the cream was based on a micro-sized emulsion [9].

Particle size is an important factor for drug permeation across paracellular and transcellular spaces. Based on the previously published report, it has been shown that the nanoparticle size formulations and positively charged particles exhibit increased cellular uptake and enhanced bioavailability. In the present study, GKNG showed a particle size of 450.14 ± 36.06 nm, zeta potential of 0.012 ± 0.003 mV, and encapsulation efficiency of 91 ± 1.8%, acceptable and optimum for stability, uniform distribution, and topical drug delivery. The FTIR result of the NG could be attributed to the ginkgolides, isoflavones, proanthocyanidins, and kaempferol binding and encapsulation in the alginate through the hydrogen of O–H binding to the oxygens of the carboxylate group [13,29,30]. The PXRD finding confirmed that binding developed between the alginate and ginkgo extract was reflected in the diffractogram of NG. The diffractogram of NG combined the nature of the alginate and ginkgo diffractograms [31,32]. Moreover, the SEM confirmed the alginate encapsulation observed in the FTIR [29]. The binding, encapsulation, and amorphicity of the NG were reflected in the shear force applied by the alginate to form the nanogel found in the particle size analysis and TEM [29,31]. After all, the alginate nature and encapsulation were observed in the diffusion, rheology, and swelling studies [13,29,31,32,33].

Considering the success of the optimized formulation, it was found that GKNG exhibited better-wound healing potential than GK gel and SA gel when comparing the effect of each sample against the untreated group. Oxidative stress is considered a limiting factor in wound healing mechanisms [23]. Increased oxidative stress is due to reduced activity of SOD and GPx and increased level of MDA that signifies lipid peroxidation [34]. Reduced activity of SOD results in impairment in the conversion of H_2_O_2_ into H_2_O and O_2_ and causes surplus production of peroxide-free radicals, causes lipid peroxidation, and MDA initiates the inflammatory cascade that initiates the inflammatory cascade causes hindrance in the wound healing [35]. Additionally, catalase causes degradation of H_2_O_2_ into H_2_O and O_2,_ and GPx acts as a scavenger of cellular peroxides. Therefore, the optimum level of SOD and GPx and reduced level of MDA accelerate the wound healing process [36]. In the present study, topical application of GKNG showed better antioxidant property than the GK gel and SA gel and showed improved wound healing potential. The findings were consistent with the previously published reports [9,26].

As discussed previously, remodeling is a critical step in wound healing. The organized deposition of collagens at the wound’s site is detrimental to the healing. The levels of COL1A1, COL4A1, and hydroxyproline directly signify the collagen deposition in the present study. The use of topical application of GKNG showed an increased level of collagens and hydroxyproline, and the findings were consistent with the previously published reports [9,26].

Apart from the remodeling, angiogenesis is another key mechanism needed for timely wound healing. The optimal density of blood capillaries ensures the maximal blood supply and supply of oxygen and the availability of ATP [37]. Angiogenesis is directly correlated with EGF and VEGF. Therefore, in the present study, we have estimated the levels of VEFG and EGF [37]. The finding showed a significant increase in their level upon treatment with the GKNG, strengthening the mechanism of wound healing by GKNG. TGF-β1 is a multifactorial cytokine needed during the entire process of wound healing [38]. The optimum level of TGF-β1 maintains the balance of the immune system and regulates the tissue repair mechanism [38].

On the one hand, TGF-β1 reduced the inflammatory cascade while assisting in the conversion of fibroblasts into myofibroblasts, as discussed previously [38]. Additionally, angiogenesis-induced by TGF-β1 also stimulates tissue repair [39]. These findings were also confirmed by a previous report where the use of a TGF-β1 inhibitor (SB431542) significantly reduced the wound healing cascade [40]. In the present study, the use of topical application of GKNG showed an increased level of TGF-β1 as compared to the GK gel and SA gel when compared with the untreated group. It hence showed improved wound healing potential, and the findings were consistent with the previously published reports [9,26].

The final step of wound healing is the contraction of the wound, and increased contraction signifies faster closure of the wound [41]. The closure of the wound is under the influence of the proliferation and remodeling phase [41]. Increased levels of antioxidant enzymes; increased COL1A1, COL4A1, and hydroxyproline levels; increased angiogenesis; and optimum level of TGF-β1 accelerate the wound contraction and closure of the wound [42]. In the present study, the use of GKNG showed improved wound contraction property as compared to GK gel and SA gel when assessed on days 5, 10, and 20, as shown in Figure 8. 

Nevertheless, histopathological analysis is considered concrete evidence of pathological damage as well as the level of improvement when the disease is treated. In the present study, the untreated group showed significant histopathological aberrations in the epidermis and dermis where acanthosis, focal hyperkeratosis, and the presence of inflammatory cells were seen. However, when the rats were treated with GKNG, improved pharmacokinetic properties were exhibited that significantly reversed the histopathological aberrations towards normal as compared to GK gel and blank SA gel.

## 4. Conclusions

In the present study, the nanocomplex gel of GK was optimized, fabricated, and characterized in terms of particle size, zeta potential, SEM analysis, and various other rheological properties needed for topical drug delivery. In addition, the developed nanocomplex gel was studied for wound healing properties. The outcome of the pharmacokinetic analysis showed improved pharmacokinetic attributes, whereas in vivo study showed improved pharmacodynamic attributes of GKNG when compared to GK gel and SA gel. In brief, GKNG exhibited statistical difference from GK gel towards increased levels of EGF, COL1A1, and GPx and against wound contraction percentage on days 5, 10, and 20. Similarly, statistical difference was found for VEGF, Hyp, SOD, and TBARS when compared to SA gel. Notably, no statistical difference was observed against GK gel and SA gel for TGF-β1 and COL4A1. Similarly, no significant difference was found against only GK gel for VEGF, Hyp, SOD, and TBARS. In conclusion, GKNG showed superior wound healing potential in terms of antioxidant markers, angiogenic markers, and improved wound contraction and showed nonsuperiority in terms of fibrotic markers. Thus, the improved pharmaceutical attributes and multifactorial mechanism of action, primarily antioxidant and angiogenesis, are responsible for the enhanced wound healing property of GKNG.

## 5. Materials and Methods

### 5.1. Drugs and Chemicals

GK extract was provided as a gift sample from Jamjoom Pharma, Jeddah, Saudi Arabia, and sodium alginate was purchased from Sigma, St. Louis, MO, USA. ELISA kits for hydroxyproline, COL1A1, COL4A1, EGF, VEGF, and TGF-β were procured from the R&D system (Tokyo Future Style Inc, Tsukuba city, Ibaraki, Japan ). In addition, various other chemicals and reagents used were of analytical grade.

### 5.2. Methods of Nanocomplex Gel Preparation

The selection of the optimum nanocomplex gel (GKNG) was based on the particle size analysis of different combinations to have the optimum nanocomplex gel. Moreover, the medium molecular weight alginate (Sigma, St. Louis, MO, USA) of a 60 mg/mL aqueous liquid solution was formed. A definite amount of 10 mg/mL GK extract was mixed with 30 mg/mL SA gel equivalent volumes, using the high-speed ultrasonication technique for 1 3 min (Table 1) using ultrasound nanomaterial dispersion instrument/homogenizer high shear emulsifier mixer probe sonicator of 20 kHz, 2000 W, and 20 mm probe diameter (Biosafer ultrasonicator, Nanjing, China) [9,13,14,31]. After that, the optimum gel was further characterized.

### 5.3. Characterization of Nanocomplex Gel

#### 5.3.1. Determination of Particle Size, PDI, Zeta Potential, and Encapsulation Efficiency

The nanoparticle extraction was performed to determine the particle size, PDI, and zeta potential by diluting 2 mL of the optimum nanocomplex gel with 2 mL of distilled water. Then, the diluted gel was mixed in a tube using a vortex for 30 s. Afterward, the mixed gel was centrifuged at 3000 rpm for 15 min for the larger particles to settle. The samples for the Zetasizer analysis (Malvern Zetasizer Nano ZSP, Worcestershire, UK) were taken from the supernatant layer [15,16,17]. Furthermore, the nanoparticle encapsulation efficiency of the optimum gel was calculated by measuring the absorbance at 365 nm using the calibration curve and Equation (2) below [17,18].

Encapsulation efficiency = (Total drug amount − Supernatant drug amount)/(Total drug amount) × 100%(2)

#### 5.3.2. FTIR Study

The 5–10 mg optimized formulation sample was used for spectral analysis through Fourier transform infrared spectroscopy (FTIR) (Thermo-Scientific, NicoletiS10, Waltham, MA, USA). The ginkgo extract (GK), alginate (A), physical mixture (PM), and the optimum nanocomplex gel (NG) samples were scanned between 500 and 4000 cm^−1^ [9,30,31,33].

#### 5.3.3. PXRD Study

Powder X-ray diffraction (PXRD) studies of the GK, A, PM, and the optimum NG were completed using Maxima XRD-7000X (Shimadzu, Kyoto, Japan). The X-rays were generated at 40 kV and 100 mA during the process by using nickel-filtered Cu Kb reduction. The scan range (2θ) ranged from 5° to 70°, and the speed was maintained at 10°/min [14].

#### 5.3.4. Structural Analysis

A transmission electron microscope (TEM, H7500, Hitachi, Tokyo, Japan) was implemented to determine the morphological characteristics of the prepared formulation GKNG. In this case, the prepared formulation was initially diluted 200 times with double distilled water, and then it was installed on a copper grid. Next, the excess sample was examined in the copper grid, and the needless sample was removed using filter paper. Next, fixed samples were stained with a phosphotungstic acid solution (0.5%) for 30 s and analyzed under TEM after drying [43]. On the other hand, scanning electron microscopy (SEM, FEI Inspect F50, FEI, Tokyo, Japan) was utilized to determine the structure and particulate distribution of the formulation in powder form. For this experiment, the liquid formulation was first dried as a film. Then, it was placed on a coverslip, and the dried sample was analyzed with a 30 kV voltage [31,33].

#### 5.3.5. Rheology Study

The rheology study of the developed formulation was carried out on a rotated rheometer (AR 2000ex, TA Instruments, New Castle, DE, USA). All the analysis was performed in triplicate at 25 °C [17]. 

#### 5.3.6. In Vitro Diffusion Study

The diffusion study was conducted based on the dialysis bag experiment in pH 6.8 up to 24 h for the optimum NG (1 mL) and the ginkgo extract diluted in 1 mL of distilled water. The studied groups were immersed in 5 mL of pH 6.8 [13]. The ginkgo extract diffusion was assessed by determining kaempferol in the ginkgo extract at 365 nm [44].

Diffusion Rate (mg/hour) = Amount diffused (mg)/Time (hour)(3)

#### 5.3.7. Swelling, Diffusibility, and Resilience Test

The approach was based on the nanohydrogel weights before and after swelling. This nanogel swelling was proportional to its diffusibility and inversely proportional to its resilience [15,17]. The optimal nanocomplex gel volume was placed in a dialysis bag (14,000 daltons cut-off, Sigma, St. Louis, MO, USA) and weighed (Wd). The enclosed nanocomplex gel was then submerged in a swelling medium with a pH of 6.8 at 37.00 ± 0.05 °C, simulating the conditions of the drug release experiment. The enclosed gel was weighed at regular intervals (Ws). The swelling weights of the nanocomplex gels were tested in triplicate. The swelling and diffusibility ratio was determined as follows:

Swelling and diffusibility ratio = (Ws − Wd)/Wd(4)

As a result, Ws is the enclosed gel weight after immersing in the media, and Wd is the enclosed nanogel weight before the start of the experiment [15,17].

### 5.4. In Vivo Study

Male Wistar rats (200–220 g, n = 50) were used in the study. The Animal Facility, King Abdulaziz University, Jeddah, Saudi Arabia, approved experimental animals. All the animals were kept in the polypropylene cage on a 12-hour light-dark cycle, and standard temperature (22.00 ± 2.00 °C) and humidity were maintained. The animal ethical committee approved experimental procedures and other activities related to animal handling of the Faculty of Pharmacy, King Abdulaziz University, Jeddah, Saudi Arabia (PH-1443-16).

#### 5.4.1. Treatment Protocol

**Figure 10:**
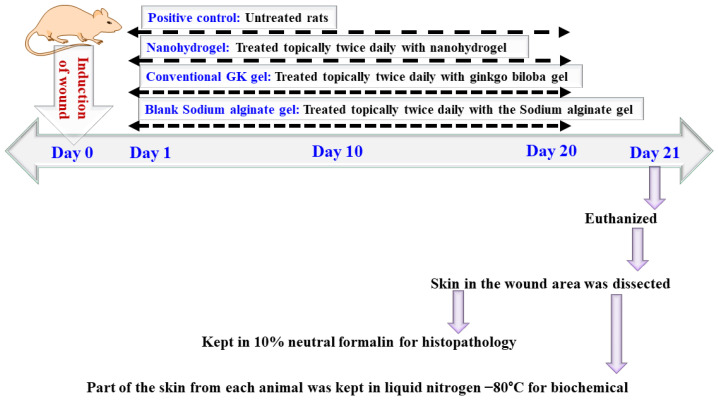
Treatment protocol used in the in vivo study.

Animals were divided into four groups (n = 4). The wound was induced by surgical excision and, on the next day onwards, topically treated with blank SA gel, GK gel, or GKNG twice daily for 20 days in comparison to the untreated group. On day 21, animals were euthanized, and the section of skin was kept in 10% formalin at −80°C for biochemical analysis, as shown in Figure 10.

#### 5.4.2. Measurement of Wound

Wound diameter was measured by using a measuring scale. After measuring the diameter, wound percentage was calculated according to the following formula:

Wound contraction = (Day zero wound diameter − Day 20 wound diameter)/Day zero wound diameter × 100(5)

#### 5.4.3. Histological Analysis

On day 21, animals were euthanized, and a section of skin was dissected and kept in 10% formalin. After 24 h, skin sections were dehydrated in an appropriate concentration by using xylene and ethanol and fixed in a paraffinized block. A thin section of 3–5 μm was made using the microtome. Then, the sections were deparaffinized, rehydrated, and stained with hematoxylin and eosin [45]. The prepared slides were analyzed blindly by the pathologist using a microscope (Nikon Eclipse Inverted Microscope, Nikon, Melville, NY, USA).

#### 5.4.4. Tissue Homogenate Preparation 

Tissues were dissected from the experimental animals, hairs were removed, the tissues were washed with ice-cooled saline water. Tissue homogenate was prepared in ice-cold phosphate buffer (pH 7.4, 4 °C). Buffer and tissue sections were mixed in the 10:1 ratio for homogenization using a tissue homogenizer. The obtained homogenate was centrifuged and used for the biochemical analysis.

#### 5.4.5. Effects of Nanocomplex Gel on Antioxidant Markers and Markers of Lipid Peroxidation

For the estimation of antioxidant markers and the markers of lipid peroxidation (MDA), obtained homogenates and subsequent supernatants were used. Firstly, the protein concentration was estimated using the Lawry method, and bovine albumin serum was the standard. Estimation of SOD, GPx, and MDA was done as per the previously published methods [46,47].

#### 5.4.6. Effects of Nanocomplex Gel on the Levels of Collagen Formation (COL1A1 and COL4A1), Hydroxyproline, and Growth Factors (EGF, VEGF, and TGF-β1)

Estimations of hydroxyproline, COL1A1, COL4A1, EGF, VEGF, and TGF-β1 concentrations were performed by using double-antibody sandwich ELISA kits (Tokyo Future Style Inc, Tsukuba city, Ibaraki, Japan). The kits were initially stored at −20 °C. First of all, samples were prepared in the dilution of 1:4, and kits were brought to the room temperature of 20–25 °C before use. Wash solution was diluted; i.e., 10 mL of wash solution was diluted with 990 mL of deionized or distilled water to prepare 1000 mL of wash solution. The rest of the components were used in their original form. Then, 100 µL of conjugate was added to each well having a test sample but not to the blank control well, mixed, and incubated for 1 h at 37 °C. After 1 h, plates were washed using prepared wash solution (350–400 µL/well/wash; soaking time 10 s and shaking time 5 s between each wash). After washing, plates were inverted and dried using absorbent paper until no moisture appeared, followed by the addition of 50 µL of substrate A and 50 µL of substrate B to each well, including blank control well. The plates were incubated for 15–20 min at 37 °C; then, 50 µL of stop solution was added to each well, and the absorbance was recorded at 450 nm using a microplate reader [26].

### 5.5. Statistical Analysis

Data were expressed as mean ± SD. One-way ANOVA followed by Tukey’s multiple comparison test was used for the statistical analysis. In statistical analysis, *p* < 0.05 was considered statistically significant. The statistical analysis was performed using GraphPad Prism 4.0 software (GraphPad Software, San Diego, CA, USA).

## Figures and Tables

**Table 1:** Illustration of the different formulation components for optimization.

Combination. No.	Ginkgo Extract. (mg/mL)	Alginate Equivalent Amount (mg/mL)	Ginkgo: Alginate Ratio	Gel Total Volume (mL)	Particle Size (nm)	PDI
1	10	30	1:3	10	450.14 ± 36.06	0.32 ± 0.12
2	20		2:3		580.25 ± 66.33	0.44 ± 0.06
3	30		3:3		860.33 ± 30.33	0.54 ± 0.91
4	40		4:3		1000.01 ± 29.33	0.59 ± 0.61

## Data Availability

The data presented in this study are available in the article.
